# Connection between Variations of the Probability Distribution of the Recurrence Time and Phases of the Seismic Activity

**DOI:** 10.3390/e25101441

**Published:** 2023-10-12

**Authors:** Elisa Varini, Renata Rotondi

**Affiliations:** 1 National Research Council of Italy, Institute for Applied Mathematics and Information Technologies, Via Corti 12, 20133 Milan, Italy; renata.rotondi@mi.imati.cnr.it; 2 ICSC National Research Centre for High Performance Computing, Big Data and Quantum Computing, Via Magnanelli 2, 40033 Casalecchio di Reno, Italy

**Keywords:** interevent time, probability distributions, probabilistic forecasting, seismic cycle, statistical seismology, statistical methods, Bayesian inference

## Abstract

The probability distribution of the interevent time between two successive earthquakes has been the subject of numerous studies for its key role in seismic hazard assessment. In recent decades, many distributions have been considered, and there has been a long debate about the possible universality of the shape of this distribution when the interevent times are properly rescaled. In this work, we aim to discover if there is a link between the different phases of a seismic cycle and the variations in the distribution that best fits the interevent times. To do this, we consider the seismic activity related to the $M_{w}$ 6.1 L’Aquila earthquake that occurred on 6 April 2009 in central Italy by analyzing the sequence of events recorded from April 2005 to July 2009, and then the seismic activity linked to the sequence of the Amatrice-Norcia earthquakes of $M_{w}$ 6 and 6.5, respectively, and recorded in the period from January 2009 to June 2018. We take into account some of the most studied distributions in the literature: *q*-exponential, *q*-generalized gamma, gamma and exponential distributions and, according to the Bayesian paradigm, we compare the value of their posterior marginal likelihood in shifting time windows with a fixed number of data. The results suggest that the distribution providing the best performance changes over time and its variations may be associated with different phases of the seismic crisis.

## 1. Introduction

The time between two successive earthquakes, referred to as recurrence or waiting or interevent time, is one of the most studied quantities describing the seismic activity; it plays an important role in seismic hazard assessment being the main component of some stochastic processes—such as renewal processes, Markov processes—which model the temporal evolution of seismic phenomena. Several probability distributions have been proposed in the literature to model the recurrence time τ, including the gamma, Weibull, lognormal, and exponential distributions, but the most remarkable result was that the shape of this distribution appeared to be universal when the times were suitably scaled by some critical indices, such as the Gutenberg–Richter *b* value, the α exponent of the Omori law and the fractal dimension $d_{f}$ of the set of earthquake epicenters [1]. In other words, the distribution F(τ) would be independent of the spatial scale and of the magnitude threshold of the observations, which expresses a hierarchical organization in time, space, and magnitude. Further studies found that scaling by the average rate λ of seismic activity—number of earthquakes per unit time—was sufficient to get approximately the same distribution in many different seismic regions [2]. A similar behavior was also obtained by Corral by fitting the density function given by

(1) $$f\left(\tau \right)=\frac{C\;\delta }{a\Gamma (\gamma /\delta )}{\left(\frac{\tau }{a}\right)}^{\gamma -1}e^{-{\left(\tau /a\right)}^{\delta }}\;\gamma ,\;\delta ,\;a>0$$

to some regional data sets [3], where *C* is the normalizing constant, *a* is a scale parameter, and γ and δ control the shape for small and large τ, respectively. This scaling function shows a power-law behavior for short times, and an exponential decrease for long times.

By simulations of the ETAS model with varying rate μ of independent events, which can be considered a proxy for regional size, Touati et al. [4] showed that the interevent time distribution is generally bimodal, and is best described as a mixture of two distributions: a gamma distribution for short waiting times between correlated events which belong to the same aftershock sequence, and an exponential distribution for longer waiting times between uncorrelated events. Completely general forms for the interevent time distribution can be obtained by resorting to a Bayesian nonparametric estimation method and considering the unknown distribution as a random measure [5].

Properties such as fractal structures and long-range correlations present in the earthquake activity have led to adopt theoretical tools of non-extensive statistical physics in the analysis of the statistical properties of some quantities describing the seismic activity in the space–time–magnitude domain. This approach is based on a generalization of the classic Boltzmann–Gibbs entropy proposed by Tsallis in 1988 [6]; by maximizing the non-extensive Tsallis entropy, a probability distribution, denoted as *q*-exponential distribution, was obtained and then successfully applied to investigate the distribution of various seismic quantities, such as magnitude ([7] and references therein), fault length [8], spatial distribution of epicenters [9], and interevent time [10]. To improve the fit of the gamma distribution by exploiting the results obtained through the *q*-exponential distribution, Michas et al. [11] used the *q*-generalized gamma distribution, borrowed from Queirós [12], which behaves as a power-law function for both short and large interevent times so as to provide a best fit when the seismicity is correlated at all timescales.

Generally, the aforementioned studies aim to obtain the best probability distribution for sets of interevent times which cover a large period, where the occurrences may involve a complex summation of triggered and/or independent events on different time scales. On the contrary, in this work, we wonder whether the probability distribution changes over time and whether these variations can be associated with different phases of the seismic process. To do this, we consider data from time windows with the same number of observations which shift at each new event; we compare the performance of some distributions chosen among the most studied ones by evaluating their posterior marginal likelihood in the Bayesian framework. We apply this procedure to two data sets related to the severe seismic sequences that hit central Italy in the last decades. The best probability distribution, i.e., the one that significantly outperforms the others, varies over time; in particular, we highlight that these changes characterize specific periods in the temporal evolution of seismic activity and therefore could be used for forecasting purposes.

## 2. Probability Distributions

Let us consider the sequence of (N+1) seismic events that occurred at times $\left\{t_{0},t_{1},\ldots ,t_{N}\right\}$, and let $𝒯={\left\{\tau_{i}\right\}}_{i=1}^{N}$ be the set of the interevent times τ between successive events defined as $\tau_{i}=t_{i}-t_{i-1}$, i=1,2,…,N. We assume that all events have a magnitude larger than, or equal to, the threshold $M_{c}$, which guarantees a sufficient degree of completeness for the data set. We present the main properties of the most studied probability distributions of the intervent time random variable.

### 2.1. Exponential Distribution

The simplest probability distribution for the interevent time is the exponential distribution with density function given by

(2) $$f_{1}\left(\tau \right)=\lambda \;e^{-\lambda \;\tau }.$$

It describes the time between events in a homogeneous Poisson point process, i.e., a process in which events occur continuously and independently at a constant average rate; hence, the exponential distribution indicates uncorrelated behavior. Its key property is that it is memoryless, that is, the probability that the waiting time τ for an event exceeds a value (t+s), conditioned on the fact that the time *t* has already passed, is equal to the original probability of exceeding *s*:

(3) $$Prob\left(\tau >t+s\;|\;\tau >t\right)=Prob\left(\tau >s\right)=\;e^{-\lambda \;s}.$$

Consequently, the exponential distribution is the only continuous probability distribution that has a constant hazard rate, equal to λ. According to Bayesian inference, the conjugate prior for the exponential distribution is the gamma distribution; hence, we consider the parameter λ to be a gamma-distributed random variable with hyperparameters $a_{0}$, $b_{0}$ so that its posterior distribution is still a gamma distribution with parameters $\left(a_{0}+N\right)$ and $\left(b_{0}+\sum_{i=1}^{N}\tau_{i}\right)$.

### 2.2. Gamma Distribution

The gamma probability density function is given by

(4) $$f_{2}\left(\tau \right)=\frac{\beta^{\alpha }}{\Gamma (\alpha )}\;\tau^{\alpha -1}\;e^{-\beta \;\tau }$$

where α is the shape parameter and β is the scale parameter, both positive. In particular, the gamma distribution models the sum of exponentially distributed random variables; that is, if we consider a sequence of events such that each interevent time follows the exponential distribution with parameter β, then the waiting time of the *n*-th event is a gamma-distributed random variable with integer shape α=n. In general, the extensive use of the gamma distribution is due to its ability to model the intertime between triggered aftershocks through its short-term scale power-law factor and the long-term scale Poissonian background activity through the exponential factor. Regarding parameter inference, since there is only the conjugate prior distribution of the scale parameter, we prefer to estimate the posterior distribution of both parameters through the Metropolis–Hastings algorithm, a stochastic simulation method of the class of Markov chain Monte Carlo (MCMC) methods. This algorithm generates a Markov chain that converges to the target distribution—in our case, the posterior distribution—using a proposal density for generating new candidate values and a method for rejecting some of the proposed values [13]. In this way, we obtain not only the parameter estimates, typically as their posterior means (average of the sampled, possibly thinned, values), but also a measure of their uncertainty as expressed through the simulated posterior distribution of each parameter.

### 2.3. *Q*-Exponential Distribution

Nonlinear dynamical systems showing fractal structures and long-range correlations are successfully studied in the framework of non-extensive statistical physics. The presence of the same properties also in seismicity [11] suggests analyzing the temporal behavior of the seismic activity through the *q*-exponential distribution:

(5) $$f_{3}\left(\tau \right)=\frac{1}{\gamma }\;{\left(1-\frac{\left(1-q\right)}{\left(2-q\right)}\;\frac{\tau }{\gamma }\right)}^{1/(1-q)},\;1<q<2$$

obtained by maximizing the non-additive Tsallis entropy $S_{q}$:

(6) $$S_{q}=k_{B}\;\frac{1-\int p^{q}\left(x\right)\;dx}{q-1}$$

under suitable constraints [6]; *q* is called the entropic index, and γ is the generalized expectation value, that is, the mean with respect to the *escort* probability distribution [14]:

(7) $$f_{q}\left(x\right)=\frac{f^{q}\left(x\right)}{\int_{0}^{+\infty }f^{q}\left(x\right)\;dx}.$$

Given two independent systems *A* and *B*, the Boltzmann–Gibbs entropy $S_{BG}$ satisfies the additive property $S_{BG}\left(A+B\right)=S_{BG}\left(A\right)+S_{BG}\left(B\right)$, whereas for the Tsallis entropy, we can verify that

(8) $$\frac{S_{q}\left(A+B\right)}{k_{B}}=\frac{S_{q}\left(A\right)}{k_{B}}+\frac{S_{q}\left(B\right)}{k_{B}}+\left(1-q\right)\frac{S_{q}\left(A\right)}{k_{B}}\;\frac{S_{q}\left(B\right)}{k_{B}}$$

i.e., $S_{q}$ is nonadditive; in particular, the cases q<1, q=1, and q>1 correspond to superadditivity (or superextensivity), additivity (extensivity), and subadditivity (subextensivity), respectively, and when q→1, $S_{q}$ recovers $S_{BG}$. Extensivity and additivity are terms often used interchangeably even though they are not equivalent because in most cases encountered in physics, additivity does imply extensivity [15]. In the Supplementary Material, we show the link between estimates of the *q*-index and specific states of the system.

For large τ, the *q*-exponential density function (5) goes to zero as a power $\tau^{-1/(q-1)}$, and it is also always bounded below by the exponential density function; hence, it is a fat-tailed distribution and, in particular, a heavy-tailed distribution, being

(9) $$\lim_{\tau \to +\infty }e^{t\tau }{\bar{F}}_{3}\left(\tau \right)=\lim_{\tau \to +\infty }e^{t\tau }{\left(1+\frac{q-1}{2-q}\frac{\tau }{\gamma }\right)}^{-(2-q)/(q-1)}=+\infty \;\forall \tau >0$$

where ${\bar{F}}_{3}\left(\tau \right)=1-F_{3}\left(\tau \right)$.

Since conjugate priors of the *q* and γ parameters with respect to the *q*-exponential function are unavailable, we again resort to Markov chain Monte Carlo methods to sample from the posterior probability distributions of the parameters after reparameterizing them as follows: θ=(2−q)/(q−1) with θ∈(0,+∞).

### 2.4. *Q*-Generalized Gamma Distribution

Substituting the exponential term in (4) with the *q*-exponential function defined as

$$\exp_{q}\left(x\right)={\left[1+(1-q)\;x\right]}^{1/(1-q)}$$

borrowed from the nonextensive statistical mechanics, one obtains the so-called *q*-generalized gamma density function, which is characterized by two power-law regimes indicating clustering effects at all time scales and both short- and long-term memory in the seismogenic process. This distribution was proposed for the first time in the financial framework by Queirós [12]; it is based on local fluctuations of the ω mean value of the gamma distributed τ variable under study. Hence, scaling the variable by its mean value, we have the conditional density function

(10) $$f\left(\tau |\omega \right)=\frac{\varphi^{\varphi }}{\omega \;\Gamma (\varphi )}{\left(\frac{\tau }{\omega }\right)}^{\varphi -1}\;\exp\left[-\frac{\varphi }{\omega }\;\tau \right]$$

where E(τ)=ω. Let us assume that ω varies over time and follows the inverse gamma distribution:

(11) $$g\left(\omega \right)=\frac{{\left(\frac{\varphi }{\lambda }\right)}^{\delta }}{\Gamma (\delta )}\;\omega^{-\delta -1}\exp\left[-\frac{\varphi }{\omega \;\lambda }\right].$$

Integrating the joint density f(τ∣ω)g(ω) with respect to ω, we obtain the marginal density function:

(12) $$h\left(\tau \right)=\frac{\lambda \;\Gamma (\varphi +\delta )}{\Gamma (\varphi )\;\Gamma (\delta )}{\left(\lambda \tau \right)}^{\varphi -1}\;{\left(1+\lambda \;\tau \right)}^{-\varphi -\delta }$$

and carrying out the changes of variables

(13) $$\lambda =\frac{\rho -1}{\xi },\;\delta =\frac{1}{\rho -1}-\varphi ,\;\alpha =\varphi -1$$

we have the *q*-generalized gamma probability density function

(14) $$f_{4}\left(\tau \right)=\frac{{\left(\rho -1\right)}^{\varphi }\;\Gamma \left(\frac{1}{\rho -1}\right)}{\xi \;\Gamma \left(\frac{1}{\rho -1}-\varphi \right)\;\Gamma \left(\varphi \right)}\;{\left(\frac{\tau }{\xi }\right)}^{\varphi -1}\;{\left[1+\frac{\rho -1}{\xi }\;\tau \right]}^{1/(1-\rho )}.$$

In the limit ρ→1, it recovers the ordinary gamma distribution. Finally, as in the case of the *q*-exponential distribution (5), we reparametrize as follows: $\eta =\frac{\left(2-\rho \right)}{\left(\rho -1\right)}$, to simplify parameter estimation through MCMC methods.

## 3. Inferential Issues

We briefly give the basic concepts on the Bayesian approach that we followed in estimation and comparison of the four models. Let us assume that the data $\tau =(\tau_{1},\tau_{2},\ldots ,\tau_{N})$ are realizations of a random variable T whose density function belongs to the parametric family F={f(τ;ψ):ψ∈Ψ}. Contrary to the frequentist approach, the parameter ψ is considered a random variable and its *prior* distribution $p_{0}\left(\psi \right)$ collects our initial beliefs about the phenomenon under study. Through the Bayes’ theorem, the information provided by the data and expressed by the likelihood $L\left(f\left(\tau \;|\;\psi \right)\right)=\prod_{i=1}^{N}f\left(\tau_{i};\psi \right)$ is combined with that in the prior distribution into the *posterior* distribution:

(15) $$p\left(\psi \;|\;\tau \right)=\frac{p_{0}\left(\psi \right)\;L\left(f\left(\tau \;|\;\psi \right)\right)}{\int_{\Psi }p_{0}\left(\psi \right)\;L\left(f\left(\tau \;|\;\psi \right)\right)\;d\psi }\;.$$

From this distribution, we obtain not only the parameter estimate, typically as the posterior mean $E_{p}\left(\psi \right)$ but also indications on its accuracy through statistical summaries such as the posterior variance or the quartiles. The computational difficulties due to the evaluation of the integral in (15), which is often multi-dimensional as in our case, can be solved by resorting to the application of Markov chain Monte Carlo (MCMC) methods, which produce a sequence of random samples from the posterior distribution (15) through which the distribution can be approximated [16]. In particular, we apply the Metropolis–Hastings (MH) algorithm, which consists of the following steps: (a) generate an initial value $\psi_{0}$ from its prior distribution $p_{0}\left(\psi \right)$ and set i=0, (b) for each iteration *i*, generate a next candidate sample $\tilde{\psi }$ from an arbitrary probability density $q(\psi |\psi_{i})$, referred to as *proposal* or *jumping* density, (c) calculate the acceptance probability

$$\alpha_{i}=min\left(1,\;\frac{L\left(f\left(\tau |\tilde{\psi }\right)\right)\;p_{0}\left(\tilde{\psi }\right)\;q\left(\psi_{i}|\tilde{\psi }\right)}{L\left(f\left(\tau |\psi_{i}\right)\right)\;p_{0}\left(\psi_{i}\right)\;q\left(\tilde{\psi }|\psi_{i}\right)}\right)\;,$$

and accept $\tilde{\psi }$ as $\psi_{i+1}$ with probability $\alpha_{i}$, or set $\psi_{i+1}=\psi_{i}$ with probability $\left(1-\alpha_{i}\right)$.

The initial values of the chain may be highly dependent on the starting value; for this, we neglect the first *k* samples and use the sequence ${\left\{\psi_{i}\right\}}_{i=k+1}^{M+k}$, for large enough values of *k* and *M*, to estimate the posterior distribution p(ψ∣τ) and to approximate the posterior marginal log-likelihood:

(16) $$\log L\left(f\left(\tau \right)\right)=\int_{\Psi }\log L\left(f\left(\tau |\psi \right)\right)\;p\;\left(\psi |\tau \right)\;d\;\psi \approx 1/M\;\sum_{j=k+1}^{M+k}\log L\left(f\left(\tau |\psi_{j}\right)\right)$$

which enables us to verify how well the fitted model f(·) is able to describe the observed data. In general, given two statistical models $f_{j}\left(\cdot |\phi \right)$ and $f_{k}\left(\cdot |\eta \right)$, we can compare them using the difference of their posterior marginal log-likelihoods $\Delta_{jk}=\log\;L\;\left(f_{j}\left(\tau \right)\right)\;-\;\log\;L\;\left(f_{k}\left(\tau \right)\right)$, and then, similar to the Bayes factor, we establish the degree of evidence in favor of the first model according to the value K of the Jeffreys scale [17,18].

Considering our four probability models in detail, we note that only the λ parameter of the exponential distribution has the gamma distribution as a conjugate prior. For each of the parameters of the other models, we choose a lognormal distribution both as a prior and as a proposal distribution in the Metropolis–Hastings algorithm. Let us consider, for instance, the parameter θ of the *q*-exponential distribution; we express our initial beliefs about it by specifying its prior distribution $\mathrm{Lognormal}({mean}_{\theta },{var}_{\theta })$, where mean and var indicate the mean and variance of the random variable θ and not the mean and variance of its logarithm, as in the common representation of the lognormal distribution. Moreover, regarding the generation of the Markov chain converging to the posterior distribution of θ, at the (i+1)-th iteration, we adopt a $\mathrm{Lognormal}\;(\theta_{i},{\left(\theta_{i}/\kappa_{1}\right)}^{2})$ as a proposal distribution for the next candidate value, where $\theta_{i}$ is the current value of the Markov chain and the value of $\kappa_{1}$ is calibrated through pilot runs so that the acceptance rate of the MH algorithm is approximately from 25% to 40%. The same applies for the other parameters.

## 4. Case Studies

To evaluate the performance of the four probability distributions of the interevent time presented in Section 2, we examine two sequences of earthquakes related to two strong seismic crises that occurred in central Italy in the last decades: the L’Aquila ($M_{w}\;6.1$) earthquake in 2009 and the Amatrice ($M_{w}\;6$)–Norcia ($M_{w}\;6.5$) earthquakes in 2016. These earthquakes have been associated with two composite seismogenic sources of the Database of Individual Seismogenic Sources (DISS, version 3.2.1) [19] that can have the potential for earthquakes up to $M_{max}\;6.5$. Both data sequences analyzed in this study are taken from the Italian National Institute of Geophysics and Volcanology (*Istituto Nazionale di Geofisica e Vulcanologia*; INGV) web services: Italian Seismological Instrumental and Parametric Database (ISIDe) working group (2016), version 1.0, accessible at http://cnt.rm.ingv.it/en/iside accessed on 6 September 2023 [20], in which the size of the events is expressed in different magnitude units, as local magnitude $M_{L}$, duration magnitude $M_{D}$, and moment magnitude $M_{w}$. We therefore applied the orthogonal regression relationships: $M_{w}=1.066\;M_{L}-0.164$ and $M_{w}=1.718\;M_{D}-1.897$ [21] to convert $M_{L}$ and $M_{D}$ to $M_{w}$ in order to construct the homogeneous data sets. The spatial extension of the areas under examination is established by taking into account the empirical relationship between the magnitude and rupture length RL—$\log_{10}\left(RL\right)=-3.22+0.69\;M_{max}$—by Wells and Coppersmith [22].

For each of the two sequences, we calculate the time between pairs of successive earthquakes in order to obtain a set of *N* observed interevent times; then, we consider time windows that consist of n=100 observations and that shift at each new event through the seismic sequence under examination. In this way, we obtain (N−n+1) data sets on which to evaluate the fitting of the probability distributions listed in Section 2 and to investigate whether the best distribution is unique or varies over time with the change of the seismic phases.

### 4.1. L’Aquila Sequence

On April 6, 2009 (01:13:40 UTC), a $M_{w}\;6.1$ shock was recorded with an epicenter at latitude 42.342 and longitude 13.380 near L’Aquila, the capital of the Abruzzo region in Central Italy. Consistent with the above criteria, we choose the rectangular area centered on the epicenter, of latitude size (41.8, 43.0) degrees and longitude size (12.8, 13.8) degrees as the study area, and we analyze the temporal period from 7 April 2005 to the end of July 2009, taking $m_{0}=2$ as the magnitude threshold to ensure the completeness of the data set, except, at most, a few hours after the main shock when temporary partial incompleteness can be observed due to the well-known difficulty in recording all the events at the beginning of the aftershock sequence, and especially those of low magnitude [23]. The main shock was preceded by a $M_{w}\;4$ foreshock on 30 March 2009 [24], and was followed by an aftershock sequence, which lasted more than a year, of which the strongest was of $M_{w}\;5.4$ occurred on 7 April 2009 [25].

Overall, we have 2725 events, that is, N=2724 interevent times through which we construct m=2625 temporal windows, each of 100 consecutive observations, shifting at each new event. To observe how the temporal distribution changes, we fit the four probability models (Section 2) to the data set associated with each time window, and then compare the pairwise differences of their posterior marginal log-likelihoods with the value K=2.3026 on the Jeffreys scale, indicating strong evidence in favor of the first model. In the case of the exponential probability density, $f_{1}\left(\tau \right)=\lambda \;\exp\left\{-\lambda \;\tau \right\}$, we adopt the conjugate Gamma(2,1) distribution as a prior distribution of the λ parameter so that the expected seismic rate is approximately 2 (time in days). For the other three probability models under examination, Table 1 reports the parameters of the prior distributions and the κ coefficients used in the proposal distributions of the MH algorithm to obtain suitable acceptance rates.

**Figure 1:**
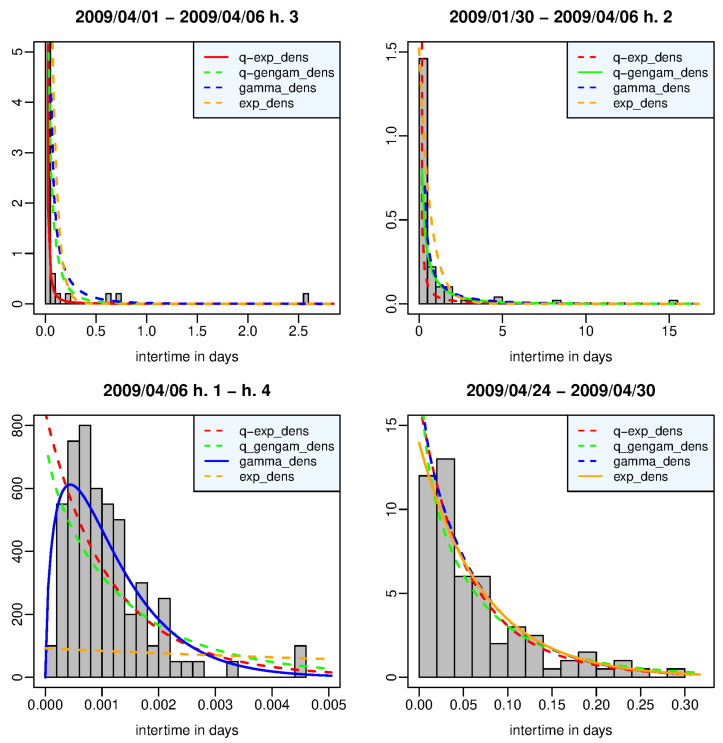
L’Aquila case—time window in which the *q*-exponential (**left top panel**), the *q*-generalized gamma (**right top panel**), the gamma (**left bottom panel**), and the exponential (**right bottom panel**) distribution provides the best fit to the data in terms of posterior marginal likelihood.

Figure 1 shows the estimated density functions and the histogram of the interevent times belonging to the time window in which each density function, represented by a solid line, provides, respectively, the best fit to the data. In particular, (a) the window in the left top panel refers to the events that occurred from 1 April 2009 up to two hours after the main shock, 90% of which are aftershocks; the *q*-exponential density function has both its largest posterior marginal likelihood and the maximum difference from the second best density function (*q*-generalized gamma). (b) Right top panel: the window contains the events from 30 January 2009 to 1 h after the main shock; the *q*-generalized gamma density function is the best model but it is not worth more than a mere mention for the evidence shown by its fit to the data with respect to the second-best density (gamma). (c) Left bottom panel: the window is all made up of aftershocks that occurred in the first three hours after the main shock; the gamma density function is the best model, and it has a decisive strength of the evidence against the second best model (*q*-exponential). (d) Right bottom panel: the window covers the period from 24 April to 30 April 2009; there is nothing more than evidence barely worthy of note in favor of the exponential density against the *q*-exponential and gamma density functions. We note that the heavy-tailed *q*-exponential distribution best describes the data set with the mass most concentrated on the left and skewed to the right, while the gamma density fits well the unimodal histogram associated with the aftershock set immediately following the main shock. The relative lack of very short interevent times is probably related to the temporary incompleteness of the catalog in that period.

**Figure 2:**
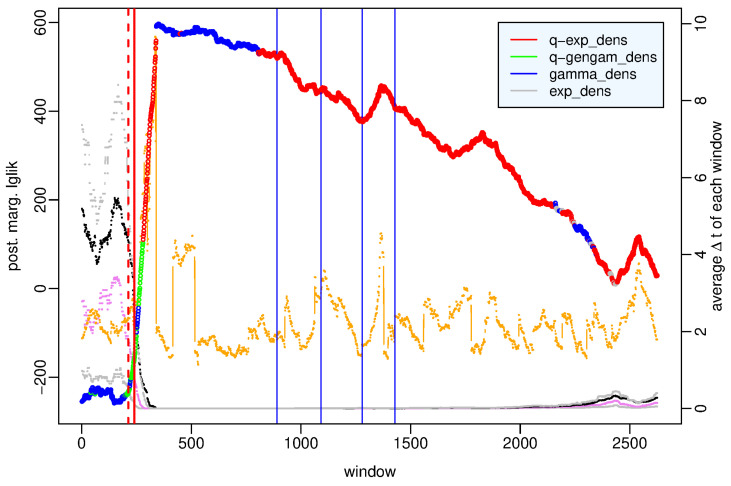
L’Aquila case—value of the posterior marginal log-likelihood of the probability distribution which provides the best fit to the data in each time window of the period (7 April 2005–31 July 2009) versus the number of the time window. Statistical summaries of the data set in each window: first and third quartile (gray dotted lines), median (violet dotted line), mean (black dotted line), skewness (orange points and line). Vertical bars indicate the occurrence time of the 30 March 2009 $M_{w}\;4$ earthquake (red dashed line), L’Aquila $M_{w}\;6.1$ earthquake (red solid line), and events of $5.0\leq M_{w}<5.5$ (blue line) respectively.

Figure 2 shows the largest value among the posterior marginal likelihoods of the four probability models at each time window; different colors highlight how the probability distribution that fits best the observations varies over time. To understand the motivations of these changes, we analyze the characteristics of each data set by showing some of their statistical summaries, such as first and third quartiles, median, mean and skewness. We remind that skewness is a measure of the asymmetry of the probability distribution. When it is positive, as in our cases, the right tail is longer, and the mean is greater than the median; more precisely, the greater the skewness, the more the distribution is left-leaning, that is, the more the observed intertimes are concentrated around short values; viceversa smaller skewness corresponds to more homogeneously distributed data where the median is closer to (to the left of) the mean.

The time windows are divided as follows: in 1745 (66%), the *q*-exponential distribution represents the best model; in 68 (2.6%), the *q*-generalized gamma distribution; in 760 (29%), the gamma distribution; and in 52 (2%), the exponential distribution. We point out that the strength of the evidence in favor of the best distribution over the second-best model is strong in only about half of the time windows, particularly for the *q*-exponential and the gamma distribution in 828 and 334 windows, respectively, concentrated in the hours after the main shock, whereas for the *q*-generalized gamma and the exponential distribution, in no window. Moreover, we note that the difference between all four models is not particularly significant in 143 (~5%, approximately from the 2300-th to 2450-th window) time windows mainly concentrated between May and June 2009; we address this issue in more detail in the Supplementary Material. For brevity, we will say hereinafter that the models are *interchangeable* when the difference between their posterior marginal log-likelihoods does not exceed the threshold K=2.3026.

We examine the characteristic features of each probability model; the *q*-exponential distribution shows very strong evidence with respect to the other distributions (the *q*-generalized gamma is the second best model) in the time windows over the main shock, that is, which include some pre-main shock event and some aftershock and during the aftershock sequence, particularly since the end of June. The gamma distribution exceeds the other distributions on the day of the main shock—6 April 2009—and is the second-best model early in the aftershock sequence, often interchangeable with the best *q*-exponential model. Substantially, the exponential distribution outperforms the other distributions with slight evidence only in a few time windows in May–June 2009.

**Figure 3:**
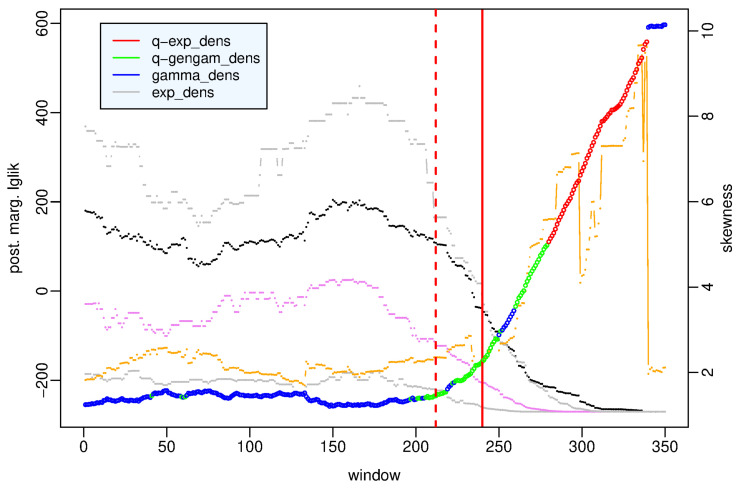
L’Aquila case—value of the posterior marginal likelihood of the probability distribution which provides the best fit to the data in the first 350 time windows covering the period (7 April 2005–6 April 2009 h. 4) versus the number of the time window. Statistical summaries of the data set in each window: first and third quartile (gray dotted lines), median (violet dotted line), mean (black dotted line), and skewness (orange points and line). Vertical red bars indicate the occurrence time of the 30 March 2009 $M_{w}\;4$ earthquake (red dashed line) and L’Aquila $M_{w}\;6.1$ earthquake (red solid line), respectively.

To better understand what happens before the main shock, in Figure 3, we zoom in on the first 350 time windows covering the period from 7 April 2005 to 6 April 2009 h. 4. The *q*-generalized gamma distribution is the best model in the period from 12 March 2009 to 6 April 2009, one hour after the main shock; that is, the period that we can denote as the preparatory phase in the seismic crisis because it includes the foreshock occurred on 30 March 2009 and is denoted by a red dotted line in Figure 3. In this period, the *q*-generalized gamma distribution is interchangeable with the gamma distribution but exceeds the other distributions with strong evidence. The role exchanges in the preceding period of background activity between April 2005 and March 2009, in which the gamma distribution is the best model, is interchangeable with the *q*-generalized gamma distribution and exceeds the other distributions with strong evidence (see the Supplementary Material).

As regards the statistical summaries, we note that the preparatory phase is characterized by a constant decrease in the mean and in the median of the data sets approximately from the beginning of 2009, while overall, the skewness increases up to 2 April 2009 and then decreases. This means that the interevent times get shorter between January and March 2009; obviously, the minimum values of the mean and median are observed during the aftershock sequence.

### 4.2. Amatrice-Norcia Sequence

In 2016–2017, the junction area of the three regions Lazio, Marche, and Umbria in Central Italy was hit by a complex sequence of destructive seismic events; on 24 August 2016 (01:36:32 UTC, latitude 42.698, longitude 13.234), an earthquake of $M_{w}\;6$ shook the city of Amatrice and caused about 300 fatalities. This shock, initially considered the main shock, later proved to be the foreshock of the $M_{w}\;6.5$ strongest shock that struck the city of Norcia on 30 October 2016 (06:40:17 UTC, latitude 42.830, longitude 13.109). The aftershock sequence lasted roughly up to July 2017 [25] and recorded four $M_{w}\;5+$ earthquakes on 18 January 2017. We consider 5062 events (N=5061 interevent times), which fall in the rectangular area of latitude size (42.3, 43.2) degrees and longitude size (12.7, 13.5) degrees and span the temporal period from January 2014 to June 2018, taking $m_{0}=2.5$ as the magnitude threshold in order to guarantee the completeness of the data set apart from the first hours following the main shock on 30 October 2016.

We investigate the behavior of the four probability distributions, given in Section 2, in m=4962 data sets, each of which obtained, by shifting at each new event, a time window constituted of 100 consecutive waiting times. First, we evaluate the posterior marginal log-likelihood of each distribution in every time window by applying the MH algorithm to estimate the posterior distribution of the model parameters; Table 1 shows the parameters of the prior distributions and the κ coefficients used in the proposal distributions of the MH algorithm to obtain suitable acceptance rates. Comparing the pairwise differences between the four posterior marginal log-likelihoods with the value K=2.3026 of the Jeffreys scale, we obtain the probability distribution with the best performance in each time window and the strength of its evidence with respect to the other distributions.

**Figure 4:**
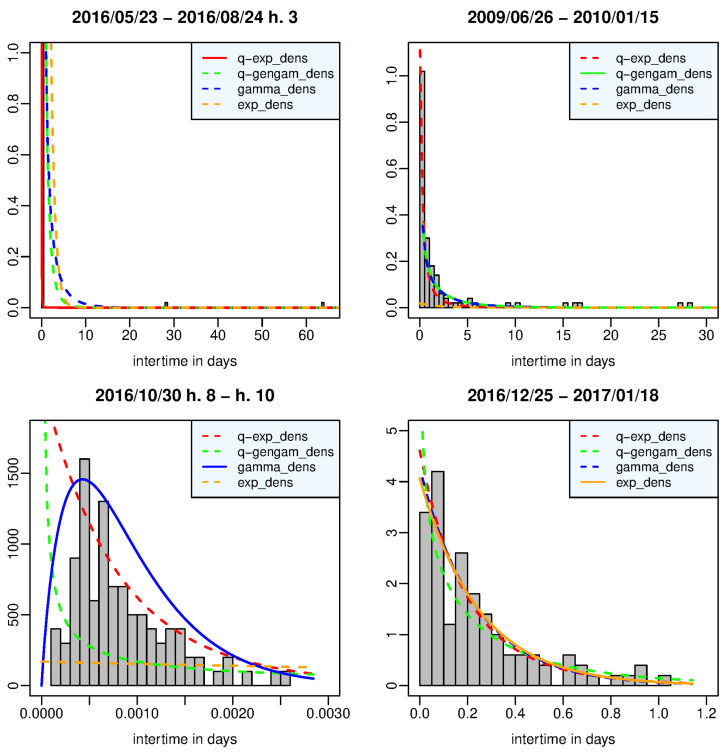
Amatrice-Norcia case—time window in which the *q*-exponential (**left top panel**), the *q*-generalized gamma (**right top panel**), the gamma (**left bottom panel**), and the exponential (**right bottom panel**) distribution provide the best fit to the data in terms of posterior marginal likelihood.

Figure 4 shows the estimated density functions and the histogram of the interevent times belonging to the time window in which each density function, represented by a solid line, provides, respectively, the best fit to the data, that is, it has its largest posterior marginal likelihood and the maximum difference from the likelihood of the second-best density function. In particular, (a) the left top panel refers to the time window which includes the first 98 aftershocks covering the two hours following the Amatrice shock, which was preceded by two waiting times of approximately 28 and 63 days; the data set is therefore very right skewed with a long right tail, and it is best described by the *q*-exponential density function, which outperforms the other distributions with decisive evidence. (b) Right top panel: the *q*-generalized gamma distribution is the best model in the time window from 26 June 2009 to 15 January 2010, which includes the final part of the L’Aquila aftershock sequence. (c) Left bottom panel: the time window includes the aftershocks that occurred between h. 8 and h. 10 of the occurrence day of the Norcia main shock (30 October 2016, 06:40:17 UTC); the gamma density function shows a decisive strength of evidence against the other probability distributions by adapting very well to the unimodal histogram of the interevent times, which are probably missing the shorter ones due to the temporary incompleteness of the catalog after the strongest event. (d) Right bottom panel: the exponential distribution is interchangeable with the gamma and *q*-exponential distribution in the time window from 25 December 2016 to 18 January 2017, when the first of the four $M_{w}\;5+$ events occurred at Capitignano.

**Figure 5:**
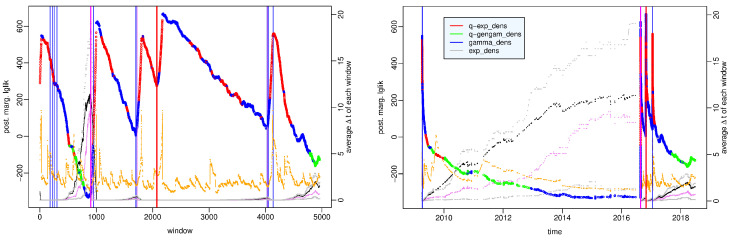
Amatrice-Norcia case—value of the posterior marginal log-likelihood of the probability distribution which provides the best fit to the data in each time window of the period (1 January 2009–31 June 2018) vs. (**left**) the number of the time window and (**right**) the time. Statistical summaries of the data set in each window: first and third quartiles (gray dotted lines), mean (black dotted line), median (violet dotted line), skewness (orange points and line). Vertical bars indicate the occurrence time of Amatrice 24 August 2016 $M_{w}\;6.0$ earthquake (magenta line), Norcia 30 October 2016 $M_{w}\;6.5$ earthquake (red line), events of $5.0\leq M_{w}<5.5$ (blue line) and of $5.5\leq M_{w}<6.0$ (violet line) respectively.

Figure 5 shows the value of the largest posterior marginal log-likelihood at each time window, and the different colors indicate which probability model this value corresponds to; the *x* axis represents the window number in the left panel and the time in the right panel. The first and third quartiles, median, mean and skewness of each data set are shown to highlight how the distribution of the observations changes over time. The time windows are divided as follows: in 1825 (36.8%), the *q*-exponential distribution represents the best model; in 360 (7.3%), the *q*-generalized gamma distribution; in 2748 (55.4%), the gamma distribution; and in 29 (0.6%), the exponential distribution. The strength of the evidence in favor of the best distribution with respect to the second-best model is strong or decisive in only about a third of the time windows, in which the outperforming distribution is especially *q*-exponential or gamma; in particular, as for the *q*-exponential, the *q*-generalized gamma and the gamma distribution in 613, 12 and 1025 windows, respectively, are essentially concentrated in the hours after the strongest shocks, whereas for the exponential distribution, in no window. The Supplementary Material provides a detailed visualization of the time windows in which the strength of the evidence in favor of a probability model is particularly significant and those in which it is not.

Let us take a more thorough look at the behavior of the various distributions. The *q*-exponential distribution is significantly the best model essentially in the first hours following the strongest earthquakes: from h. 5 to h. 8 of the day of occurrence of the L’Aquila earthquake (6 April 2009), from h. 2 to h. 3 of the day of occurrence of the Amatrice earthquake (24 August 2016), in the time windows including the first aftershocks of the Norcia earthquake (30 October 2016), and in the days of occurrence of the $M_{w}\;5+$ shocks on 18 and 19 January 2017; in the other time windows in which it has the largest log-likelihood, always during the aftershock sequences, it is interchangeable with the gamma distribution (see the Supplementary Material). The *q*-generalized gamma distribution characterizes the periods from January 2010 to November 2012 and from September 2017 to the end of our study; the difference from the other distributions has strong evidence only in January 2010, while in the other time windows, the *q*-generalized gamma density is interchangeable with the gamma density (see the Supplementary Material).

The gamma distribution has the largest value of the posterior marginal log-likelihood in most time windows—in almost half of them with strong or decisive evidence—and precisely in those covering both part of the aftershock sequences (in particular, the windows, including the first forty aftershocks following the Amatrice shock and the first hours after the main shock in Norcia) and the quiescence period from December 2012 to August 2016; in some of the first ones, the gamma model is interchangeable with the *q*-exponential model, while in the second ones, it is interchangeable with the *q*-generalized gamma model (see the Supplementary Material).

**Figure 6:**
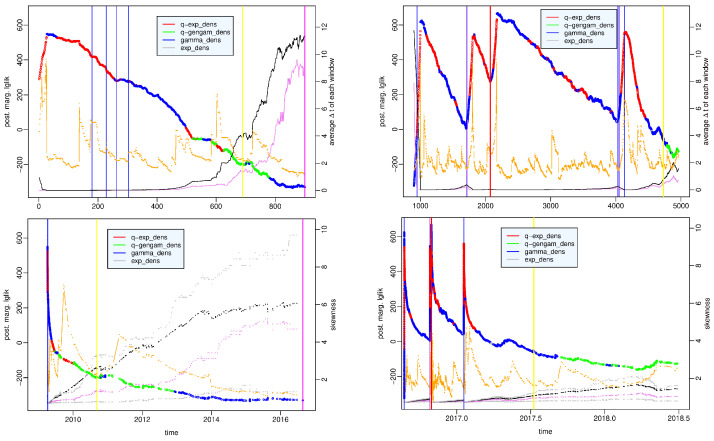
Amatrice-Norcia case—value of the posterior marginal log-likelihood of the probability distribution which provides the best fit to the data in the first 1000 time windows covering the period (1 January 2009–24 August 2016 h. 3) (**left panels**) and in the remaining time windows up to 31 June 2018 (**right panels**), versus the number of the time window (**top panels**) and versus the time (**bottom panels**). Statistical summaries of the data set in each window: mean (black dotted line), median (violet dotted line), skewness (orange points and line). Vertical bars indicate the occurrence time of Amatrice 24 August 2016 $M_{w}\;6.0$ earthquake (magenta line), Norcia 30 October 2016 $M_{w}\;6.5$ earthquake (red line), events of $5.0\leq M_{w}<5.5$ (blue line) and of $5.5\leq M_{w}<6.0$ (violet line), respectively.

In Figure 6, we distinguish the results obtained before the start of the Amatrice-Norcia seismic crisis (left panels) from those produced by the sequence of earthquakes following the Amatrice shock (right panels); the value of the largest posterior marginal log-likelihood is plotted versus the time window number in the top panels and versus time in the bottom panels. We note that the average interevent time and the likelihood are inversely correlated, i.e., the more the observed recurrence times are concentrated in short times, the greater the likelihood, or, in other words, the likelihood decreases as the seismic activity decreases. Furthermore, the gamma distribution is the best model in correspondence with the minimum values of likelihood: local minima before the shocks of greater magnitude and the absolute minimum reached on 11 February 2015. The years leading up to the onset of the Amatrice-Norcia seismic crisis are characterized by decreasing values of the log-likelihood and by the gamma distribution as the best model but with barely noteworthy evidence with respect to the *q*-generalized gamma distribution, which is the second-best model; vice versa, the *q*-generalized gamma distribution is the best model, and it is interchangeable with the gamma distribution in the years between the end of the L’Aquila aftershock sequence and the beginning of 2013. Since 2009, the average interevent time has a constantly increasing trend, which becomes almost flat starting from 2015 at the same time that the value of the median approaches that of the average.

## 5. Discussion

We investigated the probability distributions of the time between two successive earthquakes with the aim of finding out if there are links between the seismic phases and variations of the probabilistic model that best fits the data in those phases. To this end, we examined two seismic crises that hit central Italy and are related to the L’Aquila earthquake in 2009 and the Amatrice-Norcia shocks in 2016. Their retrospective analysis showed that the first crisis had a foreshock of $M_{w}\;4$ on 30 March 2009, while the second had one so strong—$M_{w}\;6$—that it was initially mistaken for the main shock. Overall, the Amatrice-Norcia sequence turned out to be more complex with greater energy release. As for the relationships between seismic phases and variations of the best probability distribution of the recurrence time, the two events share some features:

- Most of the probability densities estimated in the various time windows have a decreasing shape as an inverse power law.
- The time windows with many very short interevent times—like those in the aftershock sequences—are associated with great likelihood, while the data sets which are less concentrated around short times, typical of the quiescence period, have smaller likelihood.
- The *q*-exponential distribution outperforms the other distributions in the initial part of the aftershock sequence, and it becomes interchangeable with the gamma distribution.
- The *q*-generalized gamma distribution is associated with time intervals following aftershock sequences, such as, for example, the years from 2010 to 2012 following the aftershock sequence of L’Aquila earthquake and from September 2017 after the $M_{w}\;5+$ Capitignano shocks; in these periods, it slightly exceeds the gamma distribution. Only in the case of the L’Aquila earthquake, the *q*-generalized gamma distribution characterizes the initial phase of the activation between the fore- and the main shock.
- The gamma distribution is interchangeable with the *q*-generalized gamma distribution in the periods of low seismic activity and with the *q*-exponential distribution in part of the aftershock sequences.

Similar results have also been found in other studies, in which the phases of a seismic cycle are related to changes in the probability distribution of the magnitude [26] and of the spatial location of the epicenters [9].

Another remark can be made on the value of the log-likelihood in the case of the Amatrice-Norcia crisis. As we expect, it decreases during the aftershock sequences; however, we note that it becomes even negative just over two months after the Capitignano $M_{w}\;5+$ earthquakes, while it remains high when the same time has elapsed after the Amatrice and Norcia shocks. This could suggest that we should expect further energy releases after the Amatrice and Norcia events.

As regards the effects on the results of the possible partial incompleteness of the catalog in the few hours following a strong shock, we recall that a sensitivity analysis for the *q*-exponential distribution of the magnitude was carried out in [26] and highlighted a substantial similarity in the behavior of the entropy and of the *q*-index curves obtained from the different completeness magnitudes.

## 6. Conclusions

If, upon repeating the same analyses on different cases and in different seismotectonic contexts, the connection between changes in the probability distributions and seismic phases were confirmed, it could be hypothesized that these observations have the value of precursors of the activation level in a seismic region. The existence of the seismic precursors is still a widely open problem; but, already in 1999, Evison claimed that “despite the difficulties confronting experimentation in this field, there is growing empirical evidence that precursors exist.” [27]. Just as it is true that the most informative way to convey an earthquake prediction, including the uncertainties, is by means of probability distributions in the space–time–magnitude domain [28], it could be the same probability distributions that play the role of precursors.

We conclude by adopting Evison’s statement [27]: “Prediction is ubiquitous in science as a test of understanding: to the extent that a phenomenon is understood, it can be predicted, and vice-versa. If earthquakes were unpredictable, seismogenesis would be a closed book, research would be futile, and the earthquake would remain, in the words of Alexander McKay [29] ‘a visitation and a mistery’”.

## Figures and Tables

**Table 1:** Parameters of the prior distributions and κ coefficients used in the proposal distributions in the MH algorithm.

		L’Aquila Case			Amatrice-Norcia Case		
**Model**	**Parameters**	**Mean** ${}_{0}$	**Var** ${}_{0}$	κ	**Mean** ${}_{0}$	**Var** ${}_{0}$	κ
Gamma	α	0.04	0.01	3.0	0.8	0.15	3.0
	β	0.1	0.01	2.0	10.0	50.0	1.5
*Q*-exponential	θ	3.0	9.0	0.8	7.0	9.0	2.5
	γ	3.0	9.0	2.1	0.3	4.0	1.3
*Q*-generalized gamma	ξ	5.5	12.25	2.2	3.5	2.0	1.3
	η	6.5	6.25	2	9.0	2.5	1.6
	φ	0.7	0.04	4.0	0.7	0.02	3.5

## Data Availability

The data sets analyzed for this study can be found in the Italian Seismological Instrumental and Parametric Database (ISIDe) [20] at http://iside.rm.ingv.it (last accessed on 31 October 2021).

## Supplementary Materials

The following supporting information can be downloaded at: https://www.mdpi.com/article/10.3390/e25101441/s1.
