# Supplementary material for: Connection between Variations of the Probability Distribution of the Recurrence Time and Phases of the Seismic Activity
Source: Entropy (Basel). 2023 Oct 12;25(10):1441. doi: 10.3390/e25101441 (PMC10606842; doi:10.3390/e25101441)
Supplement: Supplementary file 1 [file entropy-25-01441-s001.zip › entropy-2626855-supplementary.pdf]

Supplementary Materials: Connection between Variations of the Probability Distribution of the Recurrence Time and Phases of the Seismic Activity

Elisa Varini [1,2†,‡] and Renata Rotondi [1,†,‡]

1. Further remarks on the results

1.1. More about the q-exponential probability distribution

The q-exponential distribution arises in the framework of the statistical mechanics by maximizing the nonextensive Tsallis entropy. The q parameter, called entropic index, is a measure of the nonextensivity of the distribution; in fact, q belongs to $\mathbb{R}^+$ and the values $q < 1$, $q = 1$, and $q > 1$ correspond to superadditivity (superextensivity), additivity (extensivity), and subadditivity (subextensivity), respectively; when $q \rightarrow 1$, the q-exponential distribution recovers the exponential and the Tsallis entropy S_q recovers the additive Boltzmann-Gibbs S_{BG} entropy. In the Figures S1 and S2 we represent the estimates of the q index in each time window highlighting with red dots those in which the q-exponential distribution is the best model for the recurrence time; we note that these windows are mainly located immediately after strong shocks and during aftershock sequences and the q-index varies between 1.084 and 1.767 in the L'Aquila case and between 1.1 and 1.822 in the Amatrice-Norcia case.

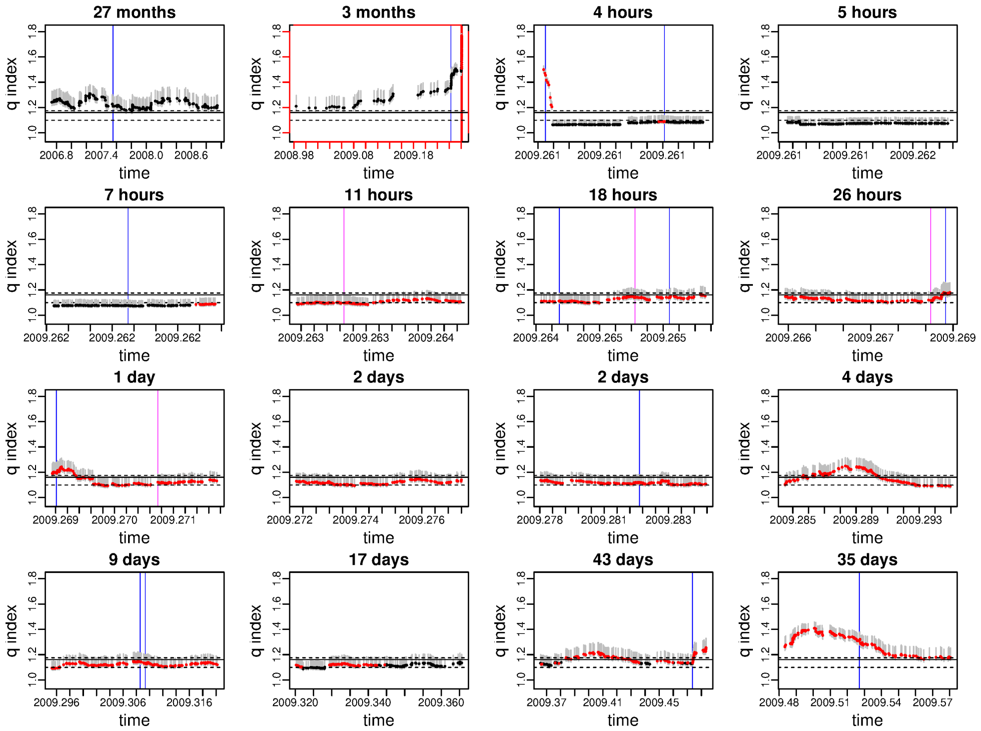

Figure S1. L'Aquila case - Estimates of the q index for each time window and first and third quartiles jointed by the gray vertical lines. The horizontal lines indicate the mean (solid line) and the first and third quartiles (dashed lines) of the estimated values. The vertical lines show the occurrence times of the events with magnitude $4 \leq M_w < 5$ (blue), $5 \leq M_w < 6$ (magenta), and $M_w \geq 6$ (red). Time is expressed in decimal format.

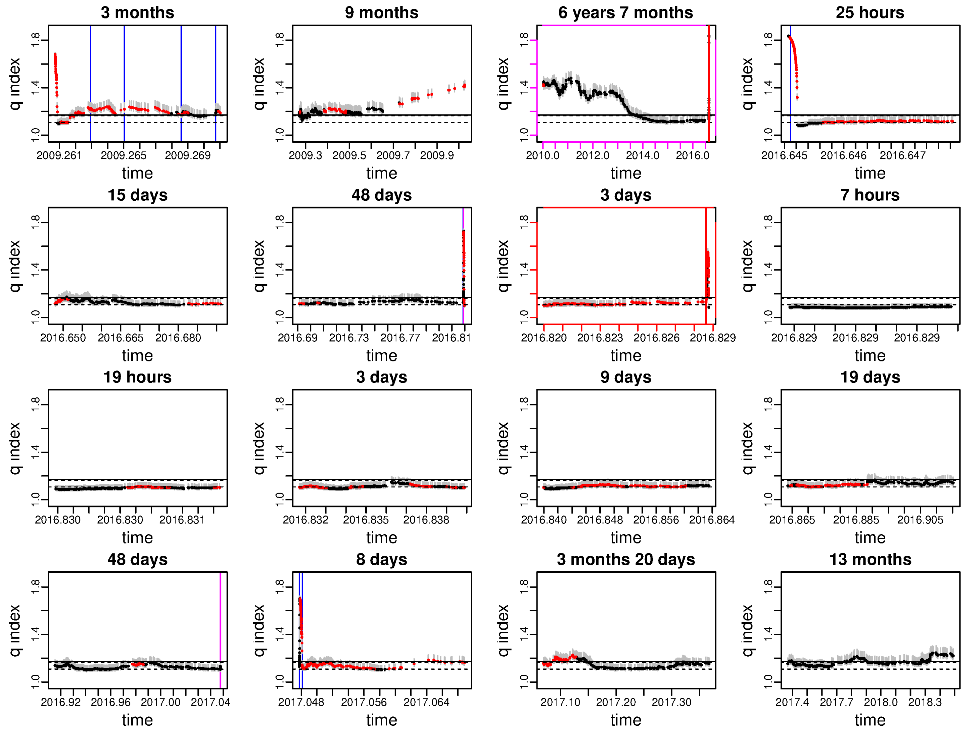

Figure S2. Amatrice-Norcia case - Estimates of the q index for each time window and first and third quartiles jointed by the gray vertical lines. The horizontal lines indicate the mean (solid line) and the first and third quartiles (dashed lines) of the estimated values. The vertical lines show the occurrence times of the events with magnitude $5 \le M_w < 5.5$ (blue), $5.5 \le M_w < 6$ (magenta), and $M_w \ge 6$ (red). Time is expressed in decimal format.

1.2. More on the degree of evidence of the best model

In addition to establishing the one that has the greatest posterior marginal likelihood as the best model among the four probability distributions of the recurrence time under examination, we also want to assign the degree of evidence of that model with respect to the others; to do this, in analogy to the Bayes factor, we compare the pairwise difference $\log B_{ij}$ between the posterior marginal log-likelihood of the model H_i and that of the model H_j with the $\mathcal{K}$ values of the Jeffreys scale, where $\log B_{ij} = \log \mathcal{L}(f_i(\boldsymbol{\tau})) - \log \mathcal{L}(f_j(\boldsymbol{\tau}))$, $i, j = 1, \ldots, 4, i \neq j$. In Table S1 we recall these values expressed in natural logarithmic scale [1].

Table S1. Values of the Jeffreys scale

$\mathcal{K}$	Evidence of H_i against H_j
0 to 1.1513	Not worth more than a bare mention
1.1513 to 2.3026	Substantial
2.3026 to 4.6052	Strong
> 4.6052	Decisive

Hereafter we denote the four probability distributions under consideration in the following way: q-exponential distribution by H_1 (red), q-generalized gamma distribution by H_2 (green), gamma distribution by H_3 (blue), and exponential distribution by H_4 (gray).

1.3. L'Aquila case

We recall that the strength of the evidence in favor of the best distribution with respect to the second best model is strong just in about half of the time windows. Figure S3

shows the value of the differences B_{1j}, $j = 2, 3, 4$ in correspondence to the time windows in which the q-exponential probability distribution is the best model; the color of the dots corresponds to the model H_j. The top panel highlights the time windows in which the evidence is more than strong in favor of H_1, while the bottom panel is divided between the values of log B_{1j} exceeding $\mathcal{K} = 1.1513$ (dotted line) which indicate substantial evidence and the values belonging to $(0, 1.1513)$ corresponding to barely noteworthy evidence against H_j. Figures S4 and S5 show analogous results for time windows where the best model is the q-generalized gamma distribution and the gamma distribution, respectively.

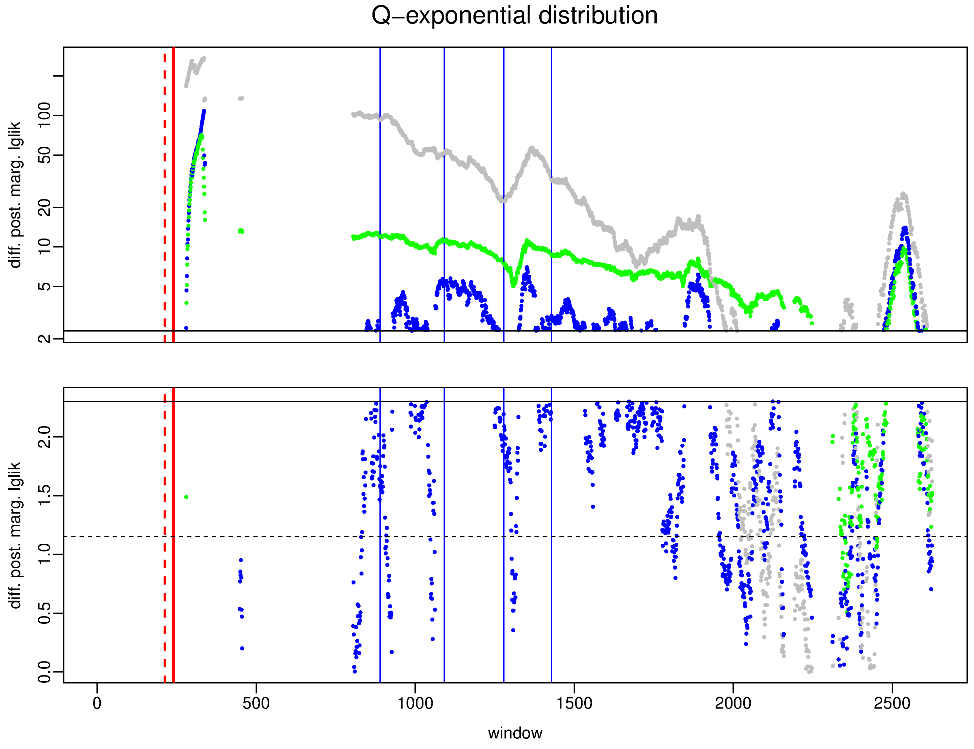

Figure S3. L'Aquila case - Differences between the posterior marginal log-likelihoods of the q-exponential and of the other distributions in the time windows in which the q-exponential distribution is the best model. The values in the top panel, in the bottom panel above and below the dotted line ($\mathcal{K} = 1.1513$) correspond to more than strong, substantial and barely noteworthy evidence in favor of the q-exponential model respectively. Vertical bars indicate the occurrence time of the 2009/03/30 M_w 4 earthquake (red dashed line) , L'Aquila M_w 6.1 earthquake (red solid line), events of $5.0 \leq M_w < 5.5$ (blue line).

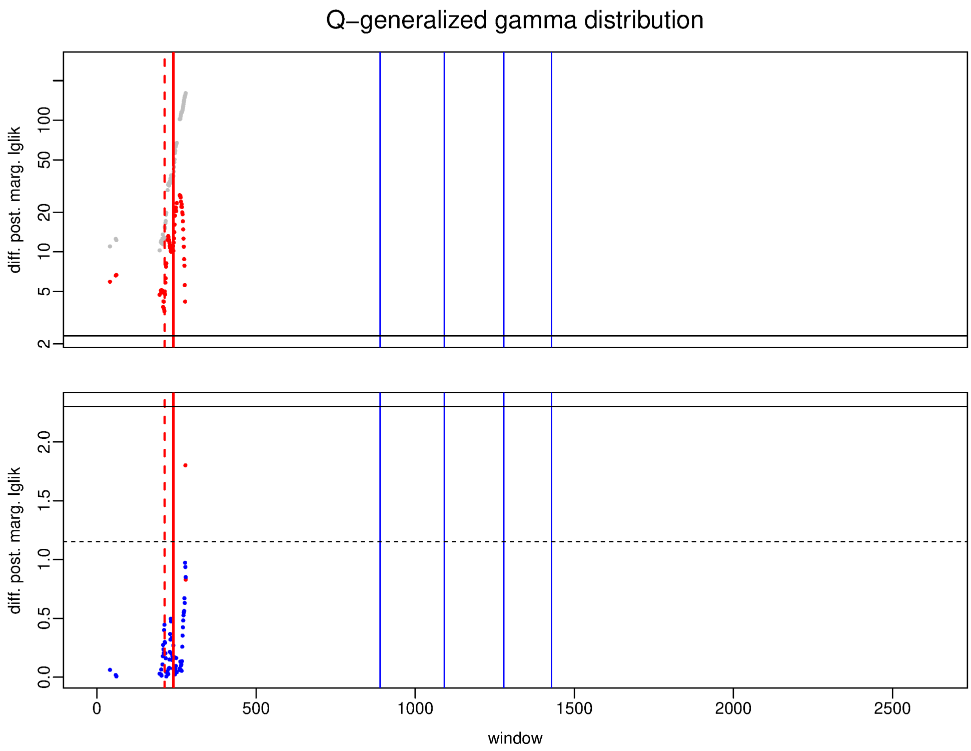

Figure S4. L'Aquila case - Differences between the posterior marginal log-likelihoods of the q-generalized gamma and of the other distributions in the time windows in which the q-generalized gamma distribution is the best model. The values in the top panel, in the bottom panel above and below the dotted line ($\mathcal{K} = 1.1513$) correspond to more than strong, substantial and barely noteworthy evidence in favor of the q-generalized gamma model respectively. Vertical bars indicate the occurrence time of the 2009/03/30 M_w 4 earthquake (red dashed line) , L'Aquila M_w 6.1 earthquake (red solid line), events of $5.0 \leq M_w < 5.5$ (blue line).

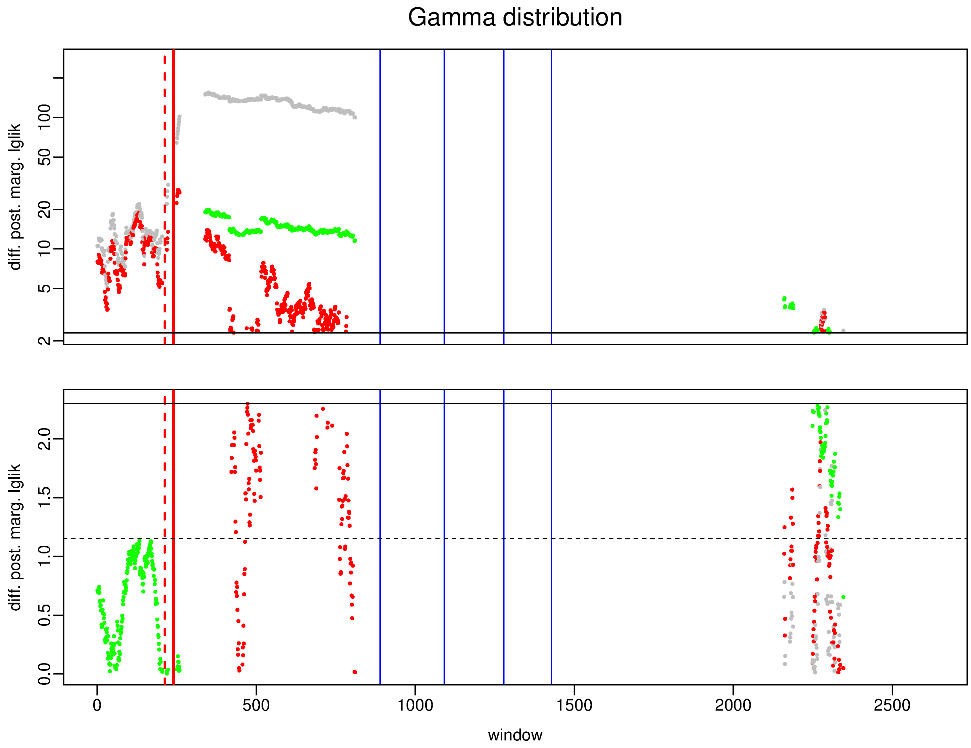

Figure S5. L'Aquila case - Differences between the posterior marginal log-likelihoods of the gamma and of the other distributions in the time windows in which the gamma distribution is the best model. The values in the top panel, in the bottom panel above and below the dotted line ($\mathcal{K} = 1.1513$) correspond to more than strong, substantial and barely noteworthy evidence in favor of the gamma model respectively. Vertical bars indicate the occurrence time of the 2009/03/30 M_w 4 earthquake (red dashed line), L'Aquila M_w 6.1 earthquake (red solid line), events of $5.0 \leq M_w < 5.5$ (blue line).

2. Amatrice-Norcia sequence

We recall that the strength of the evidence in favor of the best distribution with respect to the second best model is strong or decisive just in about a third of the time windows, in which the outperforming distribution is especially q-exponential or gamma. As in the case of L'Aquila sequence Figures S6, S7, S8 show the values of $\log B_{ij}$, $i = 1, 2, 3$, in correspondence of the time windows in which the best model H_i has more than strong (top panel), substantial (upper part of the bottom panel) and barely noteworthy evidence (lower part of the bottom panel) against H_j, $i \neq j$.

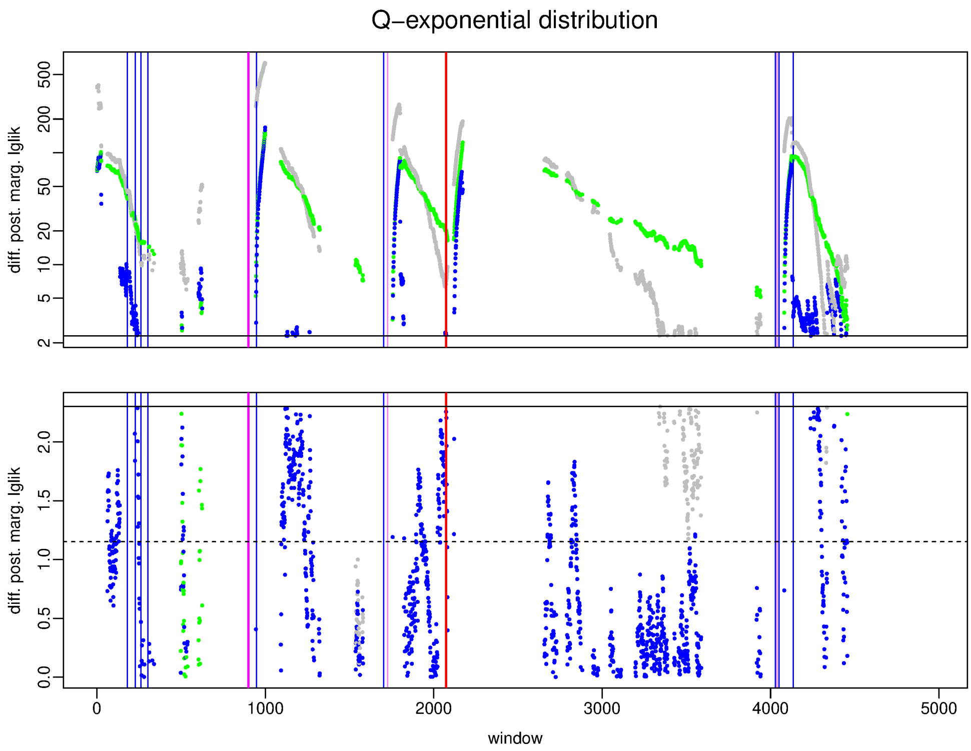

Figure S6. Amatrice-Norcia case - Differences between the posterior marginal log-likelihoods of the q-exponential and of the other distributions in the time windows in which the q-exponential distribution is the best model. The values in the top panel, in the bottom panel above and below the dotted line ($\mathcal{K} = 1.1513$) correspond to more than strong, substantial and barely noteworthy evidence in favor of the q-exponential model respectively. Vertical bars indicate the occurrence time of Amatrice 2016/08/24 M_w 6.0 earthquake (magenta line), Norcia 2016/10/30 M_w 6.5 earthquake (red line), events of $5.0 \leq M_w < 5.5$ (blue line) and of $5.5 \leq M_w < 6.0$ (violet line).

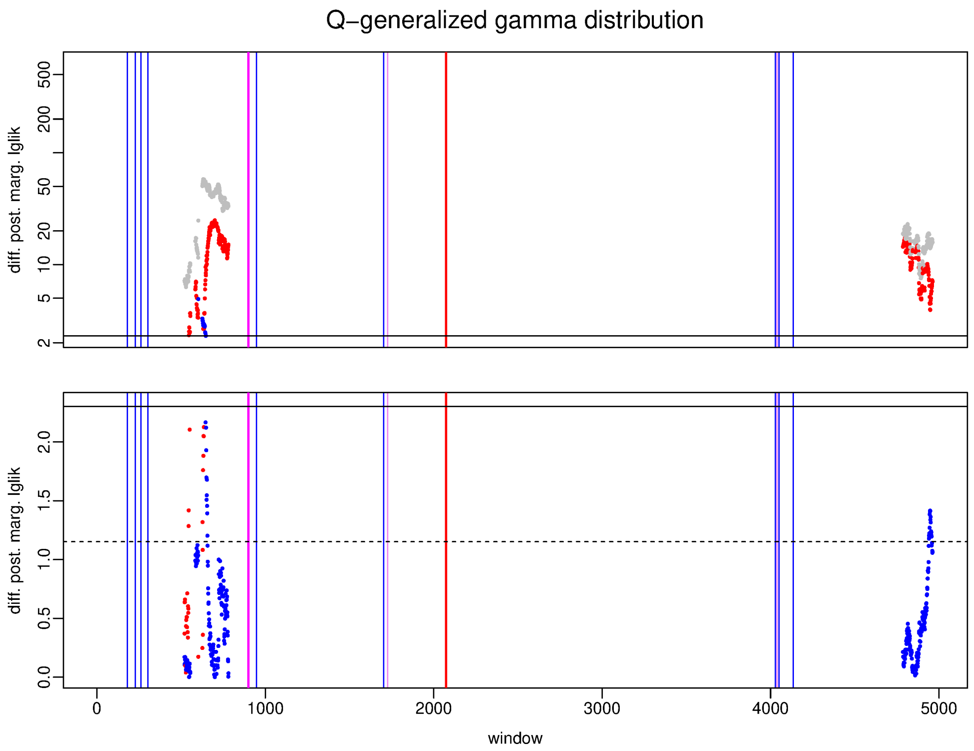

Figure S7. Amatrice-Norcia case - Differences between the posterior marginal log-likelihoods of the q-generalized gamma and of the other distributions in the time windows in which the q-generalized gamma distribution is the best model. The values in the top panel, in the bottom panel above and below the dotted line ($\mathcal{K} = 1.1513$) correspond to more than strong, substantial and barely noteworthy evidence in favor of the q-generalized gamma model respectively. Vertical bars indicate the occurrence time of Amatrice 2016/08/24 M_w 6.0 earthquake (magenta line), Norcia 2016/10/30 M_w 6.5 earthquake (red line), events of $5.0 \leq M_w < 5.5$ (blue line) and of $5.5 \leq M_w < 6.0$ (violet line).

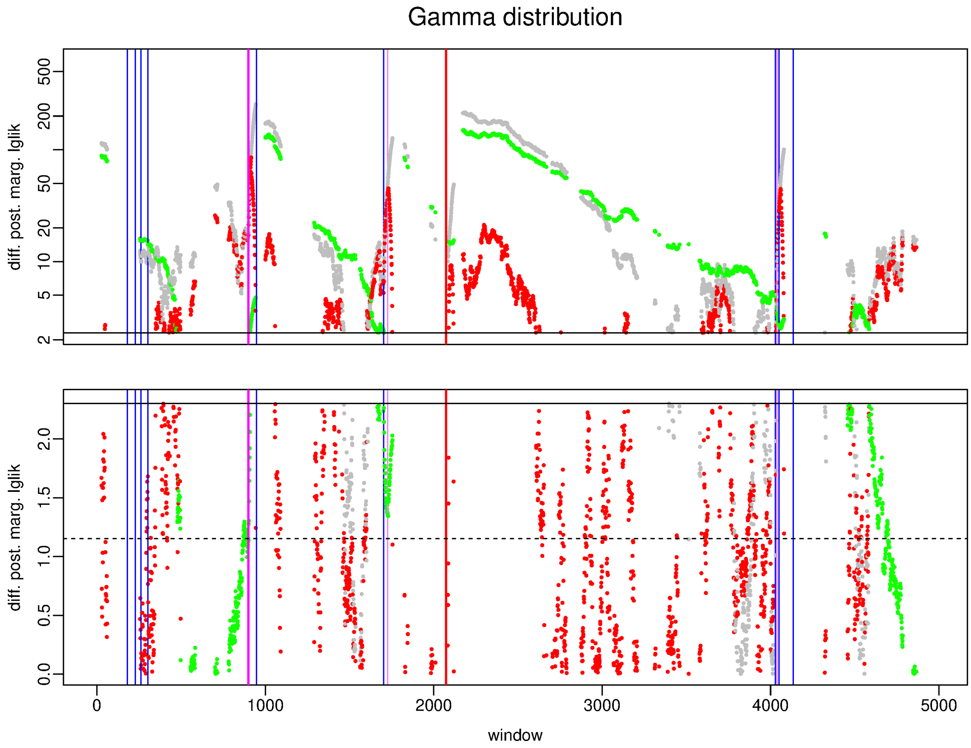

Figure S8. Amatrice-Norcia case - Differences between the posterior marginal log-likelihoods of the gamma and of the other distributions in the time windows in which the gamma distribution is the best model. The values in the top panel, in the bottom panel above and below the dotted line ($\mathcal{K} = 1.1513$) correspond to more than strong, substantial and barely noteworthy evidence in favor of the gamma model respectively. Vertical bars indicate the occurrence time of Amatrice 2016/08/24 M_w 6.0 earthquake (magenta line), Norcia 2016/10/30 M_w 6.5 earthquake (red line), events of $5.0 \leq M_w < 5.5$ (blue line) and of $5.5 \leq M_w < 6.0$ (violet line).

References

1. Kass, R.E.; Raftery, A.E. Bayes factor, **1995**, *J. Am. Stat. Ass.*, **90**, 430, 773–795, doi=10.2307/2291091.
